# Cross-Sectional Survey to Assess Health-Care Workers’ Grief Counseling for Bereaved Families of COVID-19 Victims in Wuhan, China

**DOI:** 10.1017/dmp.2021.132

**Published:** 2021-04-30

**Authors:** Xudong Gao, Zhimin Wang, Chan Kong, Hongru Fan, Juan Zhang, Jing Wang, Lingling Tan, Jinyao Wang

**Affiliations:** 1 College of Medicine and Health Science, Wuhan Polytechnic University, Wuhan, China; 2 Department of Nursing, Second Affiliated Hospital of University of South China, Hengyang, China; 3 General Medical Department, Tongji Hospital, Tongji Medical College, Huazhong University of Science and Technology, Wuhan, China; 4 Department of Cardiovascular Diseases, First Hospital of Wuhan, Huazhong University of Science and Technology, Wuhan, China; 5 Department of Nursing, Second Hospital of Wuhan, Huazhong University of Science and Technology, Wuhan, China; 6 Department of Neurosurgery, Fourth Hospital of Wuhan, Huazhong University of Science and Technology, Wuhan, China; 7 Department of Nursing, Second Affiliated Hospital of University of South China, Hengyang, China; 8 Public Health Teaching Center, Department of Medicine, Shanxi Datong University, Datong, China

**Keywords:** bereavement, grief counseling, COVID-19, health care workers

## Abstract

**Objective:**

This research aimed to examine health-care workers’ grief counseling for bereaved families of coronavirus disease 2019 (COVID-19) victims in China. Our research may provide a new opportunity to stimulate development of grief counseling in China.

**Methods:**

A cross-sectional survey was conducted with 724 health-care workers selected by convenience sampling from 7 hospitals in Wuhan. Data collection tools included a sociodemographic questionnaire, the skills of grief counseling scale (SGCS), and the attitudes of grief counseling scale (AGCS).

**Results:**

The average SGCS score was 18.96 ± 4.66, whose influencing factors consisted of sense of responsibility, frequency of contact with bereaved families, and relevant training (*P* < 0.05). The average AGCS score was 33.36 ± 8.70, whose influencing factors consisted of other grief counseling skills, communication skills, education background, and relevant training (*P* < 0.05).

**Conclusions:**

The skills and attitudes toward grief counseling among health-care workers combating COVID-19 were at a lower level in Wuhan, China, indicating the need to build a comprehensive grief counseling system, establish a standardized training course, and strengthen the popularization of grief counseling services to the public.

Coronavirus disease 2019 (COVID-19) has created a mortality shock around the world, it may create a second wave of psychosocial problems tied to bereavement.^1^ First reported in Wuhan, the capital city of Hubei, China, in December 2019, COVID-19 then spread across China.^2,3^ Particularly, the death toll of the COVID-19 in Wuhan increased sharply from January to April in 2020.^4,5^ As of January 9, 2021, the death toll from COVID-19 in Chinese mainland was 4798, and in Wuhan, it was 3869.^6^ Owing to the sudden infection and rapid deterioration of patients affected by the illness, many Chinese families were unprepared to accept the death of a family member.^7^ When the COVID-19 patients passed away, many bereaved families encountered various mental and psychological problem, for instance depression, anxiety, and complicated grief.^8^ More seriously, some of those grieving the loss of family members due to COVID-19 engaged in self-harm or violence against other people.^9^ There were also reports of a series of conflicts between bereaved families and health-care workers in Wuhan.^9^

Grief counseling plays an important role in postdisaster psychological service.^10,11^ It is a service for those who have suffered a significant loss.^12,13^ The target groups of grief counseling mainly included adults who have lost their husband or wife, pregnant women who have lost their fetuses, parents who have lost their children, and children who have lost their parents.^14^ In general, health-care workers are the first to contact bereaved families, and the grief counseling they provide can relieve the mental and psychological problems of bereavement to a considerable degree.^15^ Grief counseling was first introduced in mainland China as a result of a great earthquake that occurred in Sichuan Province, China, in 2008.^16^ However, to date, grief counseling is still not included in the medical curriculum system in China.^17^ Therefore, many Chinese health-care workers have not received relevant systematic courses.^18^ When Chinese health care workers encounter the death of patients, they also feel sympathy, but they often are unaware of what measures can be taken to support bereaved families.^19^

To date, research on healthcare workers combating COVID-19 have mainly focused on mental health, manpower management, and infection prevention.^20–22^ As far as we know, there is no research investigating health-care workers’ grief counseling service for bereaved families of COVID-19 victims in China. Thus, this research was conducted to fill this gap. It is hoped that our research will provide an opportunity to stimulate all-round development of grief counseling in China.

## Methods

### Study Design and Participants

We launched a cross-sectional survey using an electronic questionnaire. Taking into consideration data collection availability and quality, health-care workers from 7 hospitals in Wuhan were investigated by convenience sampling. The electronic questionnaires were distributed to the health-care workers’ cell phones through their medical service departments and nursing departments. Participants were informed that they needed to meet the inclusion criteria for research enrollment, which included: (1) nurses or doctors who had worked on the frontline against COVID-19 in Wuhan, (2) those who had received bereaved families of COVID-19 victims, and (3) those who voluntarily joined this research. All participants received information about the research aim and were informed that participation was voluntary, and results would be kept confidential. Informed consent was obtained before conducting each on-line survey. This study anonymously investigated the status of grief counseling among health-care workers, and did not involve human biomedical research. Therefore, this research was ruled exempt by the Institutional Review Board of Wuhan Polytechnic University. In total, 785 health-care workers completed our questionnaire from February 2020 to May 2020. In total, 61 questionnaires lacking indispensable information were excluded, and finally 724 questionnaires were collected.

## Survey Questionnaire

The questionnaire contained 3 parts, and it took the health-care workers approximately 7 min to complete.

### Sociodemographic Characteristics

Sociodemographic characteristics were gathered on gender (male, female), occupation (nurse, doctor), work experience (1-5 y; 6-10 y; 11-45 y), education background (bachelor, master, doctorate), marital status (unmarried, married), religion (religious, nonreligious), professional title (junior title, intermediate title, senior title/deputy senior title), frequency of contact with bereaved families (low, middle, high), training related to grief counseling (untrained, trained), and bereavement experience (without, with).

### Skills of Grief Counseling Scale

Zhang established the skills of grief counseling scale (SGCS) in 2017.^23^ The Cronbach’s α coefficient is 0.851, the content validity index is 0.871, and the reliability index is 0.834.^19,23^ SGCS includes 2 parts: communication skills (3 items) and other grief counseling skills (5 items). All items were answered on a 4-point Likert scale ranging from 1 (totally disagree) to 4 (totally agree). Higher scores reveal increased competency in grief counseling for bereaved families.

### Attitudes of Grief Counseling Scale

Zhang established the attitudes of grief counseling scale (AGCS) in 2017.^23^ The Cronbach’s α coefficient is 0.935, the content validity index is 0.892, and the reliability index is 0.915.^19,23^ AGCS includes 3 parts: sense of responsibility (3 items), willingness to receive relevant training (3 items), and enthusiasm for grief counseling (4 items). All items were answered on a 5-point Likert scale ranging from 1 (totally disagree) to 5 (totally agree). The higher the score, the more positive the health-care workers’ attitude toward bereaved families.

### Statistical Methods

We used IBM SPSS Statistics 25.0 for statistical analysis. The data normal distribution was tested by Kolmogorov Smirnov analysis. After testing, SGCS and AGCS were both normally distributed. Multicollinearity was measured by the variance inflation factor (VIF). After testing, values of VIF were all lower than 10, so there is no multicollinearity. The comparison of different sociodemographic characteristics of health-care workers’ skills and attitudes toward grief counseling were analyzed by univariate analysis. The correlations between grief counseling skills and attitudes toward grief counseling were analyzed using Pearson correlation analysis. Multiple-factor analysis of skills of grief counseling and attitudes of grief counseling were, respectively, analyzed using multiple regression analysis. For all tests, values of *P* < 0.05 were considered statistically significant.

## Results

### Skills and Attitudes of Grief Counseling Among Health-Care Workers

Table 1 showed health-care workers’ skills and attitudes of grief counseling toward bereaved families of COVID-19 victims in Wuhan. The average SGCS score was 18.96 ± 4.66, with a mean score rate of 59.2%. The maximum score of SGCS was 30, and the minimum was 9. The total point of AGCS was 33.36 ± 8.70, with a mean score rate of 66.6%. The maximum score of AGCS was 46, and the minimum was 15.

**Table 1.** Scores of skills and attitudes of grief counseling among health-care workers during the COVID-19 pandemic (*n* = 724)

Item	Total points (Mean ± *S*)	Average points of items (Mean *± S*)	Score rates (%)
SGCS	18.96 ± 4.66	2.37 ± 0.58	59.2
Communication skills	9.04 ± 2.33	3.01 ± 0.77	75.2
Other grief counseling skills	9.92 ± 2.92	1.98 ± 0.58	49.5
AGCS	33.36 ± 8.70	3.33 ± 0.87	66.6
Sense of responsibility	11.19 ± 2.69	3.73 ± 0.89	74.6
Enthusiasm for grief counseling	12.40 ± 3.63	3.10 ± 0.90	62.0
Willingness to receive training	9.78 ± 3.17	3.26 ± 1.05	65.2

### Comparison of Different Sociodemographic Health-Care Workers’ Skills and Attitudes of Grief Counseling

As indicated in Table 2, health care workers’ SGCS scores were significantly associated with frequency of contact with bereaved families and relevant training (*P* < 0.05). Health-care workers’ AGCS scores were significantly associated with education background, professional title, frequency of contact with bereaved families, and relevant training (*P* < 0.05).

**Table 2.** Comparison of different sociodemographic health-care workers’ skills and attitudes of grief counseling during the COVID-19 pandemic (*n* = 724)

Factor	No.	(%)	SGCS Total points (Mean ± SD)	F	P	AGCS Total points (Mean ± S)	F	P
Gender				0.148	0.701		2.527	0.112
Male	203	28.0%	18.86 ± 4.62			34.19 ± 8.05		
Female	521	72.0%	19.01 ± 4.68			33.04 ± 8.92		
Occupation				0.037	0.848		0.958	0.328
Nurse	390	53.9%	18.99 ± 4.51			33.07 ± 8.62		
Doctor	334	46.1%	18.93 ± 4.85			33.71 ± 8.78		
Work experience				2.263	0.105		2.729	0.066
1-5	266	36.7%	18.50 ± 4.97			32.69 ± 8.72		
6-10	259	35.8%	19.35 ± 4.55			33.15 ± 8.36		
11-45	199	27.5%	19.09 ± 4.36			34.55 ± 9.02		
Education background				2.387	0.093		18.058	<0.001
Bachelor	383	52.9%	18.68 ± 4.33			31.60 ± 8.88		
Master	204	28.2%	19.56 ± 5.17			34.97 ± 8.76		
Doctorate	137	18.9%	18.86 ± 4.73			35.91 ± 6.84		
Marital status				3.568	0.059		0.349	0.555
Unmarried	340	47.0%	19.31 ± 4.83			33.57 ± 8.93		
Married	384	53.0%	18.66 ± 4.50			33.18 ± 8.49		
Religion				0.028	0.867		0.030	0.862
Nonreligious	606	83.7%	18.98 ± 4.51			33.39 ± 8.70		
Religious	118	16.3%	18.90 ± 5.43			33.24 ± 8.72		
Professional title				2.413	0.090		7.910	<0.001
Junior	335	46.3%	18.74 ± 4.81			32.36 ± 9.15		
Intermediate	326	45.0%	18.97 ± 4.58			33.71 ± 8.34		
Senior/deputy senior	63	8.7%	20.14 ± 4.18			36.92 ± 6.89		
Frequency of contact with bereaved families				78.948	<0.001		26.245	<0.001
Low (< 5 times)	204	28.2%	15.87 ± 4.26			30.34 ± 9.17		
Middle (5-10 times)	309	42.7%	19.79 ± 4.38			33.34 ± 8.55		
High (>10 times)	211	29.1%	20.75 ± 3.96			36.32 ± 7.36		
Relevant training				82.753	<0.001		44.587	<0.001
Untrained	474	65.5%	17.88 ± 4.67			31.84 ± 8.75		
Trained	250	34.5%	21.02 ± 3.90			36.25 ± 7.84		
Bereavement experience				0.010	0.920		0.248	0.618
Without	382	52.8%	18.95 ± 4.88			33.21 ± 8.88		
With	342	47.2%	18.98 ± 4.42			33.54 ± 8.50		

### Correlation Between Skills of Grief Counseling and Attitudes of Grief Counseling Among Health-Care Workers

The total SGCS score and its dimensions were positively correlated with AGCS and its dimensions as shown in Table 3 (*P* < 0.01). Better grief counseling skills of health-care workers corresponded with better attitudes.

**Table 3.** Relevance between skills of grief counseling and attitudes of grief counseling among health-care workers during the COVID-19 pandemic (*n* = 724)

Item	AGCS	Sense of responsibility	Enthusiasm for grief counseling	Willingness to receive training
SGCS	0.461	0.466	0.404	0.404
Communication skills	0.403	0.406	0.363	0.344
Other grief counseling skills	0.414	0.420	0.356	0.370

#### Multiple-Factor Analysis of Skills of Grief Counseling Among Health-Care Workers

The main influence factors of skills of grief counseling consisted of sense of responsibility, frequency of contact with bereaved families, and training, as shown in Table 4. Therefore, the negative factors were lack of responsibility, low frequency of contact with bereaved families, and no relevant training.

**Table 4.** Multiple-factor analysis of skills of grief counseling among health-care workers during the COVID-19 pandemic (*n* = 724)

Independent variable	Regression coefficient	Standardized regression coefficient	*t*	*P*
Constant	6.809	—	9.816	<0.001
Sense of responsibility	0.649	0.374	11.649	<0.001
Frequency of contact with bereaved families	1.475	0.239	6.976	<0.001
Relevant training	1.438	0.147	4.322	<0.001

Annotation: R^2^ = 0.316, adjusted R^2^ = 0.313, F = 110.701, *P* < 0.05.

#### Multiple-Factor Analysis of Attitudes of Grief Counseling Among Health-Care Workers

The main influence factors of attitudes toward grief counseling consisted of other grief counseling skills, communication skills, education background, and training, as shown in Table 5. Therefore, the negative factors were lack of other grief counseling skills, lack of communication skills, low education levels, and no relevant training.

**Table 5.** Multiple-factor analysis of attitudes of grief counseling among health-care workers during the COVID-19 pandemic (*n* = 724)

Independent variable	Regression coefficient	Standardized regression coefficient	*t*	*P*
Constant	12.254	—	8.800	<0.001
Other grief counseling skills	0.669	0.225	5.502	<0.001
Communication skills	0.945	0.254	6.482	<0.001
Education background	2.127	0.190	5.893	<0.001
Relevant training	1.779	0.097	2.836	0.005

Annotation: R^2^ = 0.259, adjusted R^2^ = 0.254, F = 62.677, *P* < 0.05.

## Discussion

In any epidemic or pandemic, bereaved families are often ignored. At present, the COVID-19 pandemic in China has been basically controled, but the psychological trauma of bereaved families may linger for an extensive period of time. This is the first research to analyze health-care workers’ grief counseling toward bereaved families of COVID-19 victims in China.

### Skills of Grief Counseling Among Health-Care Workers

The results of our survey showed that the average SGCS score among health-care workers was 18.96 ± 4.66, with a mean score rate of 59.2%. This indicated that health-care workers’ grief counseling skills need to be improved urgently. The following 2 reasons may explain this phenomenon. First, it is likely that a large number of Chinese health-care workers have not received systematic grief counseling training.^24^ Unlike some developed countries that have established a complete grief counseling system, grief counseling is still in its preliminary stage in China.^25^ As mentioned before, grief counseling has not been included in the medical curriculum in China.^17^ Nevertheless, it is gratifying that more and more Chinese hospitals have realized the importance of grief counseling and have started providing relevant training for their health-care workers in recent years.^26^ Second, some of the health-care workers on the frontline of pandemic fight had limited experience of receiving bereaved families in their previous work, which also resulted in their poor grief counseling skills. During the COVID-19 outbreak period, health-care workers from almost all departments were mobilized to deal with issues of insufficient human resources in Wuhan.^27,28^ Generally speaking, health-care workers from the emergency department, intensive care unit, and oncology department are more likely to contact bereaved families. Hence, a large proportion of health-care workers lack sufficient experience in receiving bereaved families.

### Attitudes of Grief Counseling Among Health-Care Workers

The average AGCS score among health-care workers was 33.36 ± 8.70, with a score rate of 66.6%, indicating that the attitude of grief counseling among health-care workers toward bereaved families of COVID-19 victims was not very positive. The following 3 reasons may account for this result. First, job burnout among health-care workers may have an impact on their attitudes toward grief counseling. An emergency hierarchical medical system was established in Wuhan during the first quarter of 2020.^29^ A total of 51 hospitals were transformed into COVID-19–designated hospitals by the Wuhan municipal government; these hospitals were responsible for treating patients with severe clinical symptoms.^30,31^ As the pandemic progressed, tens of thousands of patients flooded into these COVID-19–designated hospitals, leading to severe shortages in hospital personnel.^28^ Many health-care workers in Wuhan had to continuously work overtime, resulting in different degrees of job burnout.^32,33^ Previous research demonstrated that job burnout of health-care workers may cause compassion fatigue.^34^ Thus, it is reasonable to infer that a huge workload may have weakened the compassion of health-care workers toward bereaved families to some degree. Second, it is possible that psychological issues among health-care workers affected their service attitudes. A vast number of health-care workers suffered from psychological issues, such as depression, fear, and anxiety in combating the COVID-19 pandemic.^35,36^ While health-care workers struggled with severe acute respiratory syndrome, their psychological issues had a negative impact on their work attitude.^37^ These health-care workers afflicted by various psychological problems engaged in a series of unprofessional behaviors and poor work performance, such as decreased punctuality, less contact with patients, and reduced working time.^37^ Last, bereaved families’ behaviors will also affect the of health-care workers’ attitudes toward grief counseling. Among Chinese, death is regarded as a taboo topic whose mention is usually avoided in social interactions. When relatives pass away, Chinese either bear the grief alone or seek comfort from family members.^38^ Because bereaved families in China rarely seek help from health-care workers, health-care workers take for granted that they are not responsible for offering grief counseling service to the bereaved.

### Factors Affecting Skills of Grief Counseling Among Health-Care Workers

Our research demonstrated that the potential influencing factors of grief counseling skills comprised of sense of responsibility, frequency of contact with bereaved families, and relevant training. To begin with, we found that possessing a strong sense of responsibility was closely related to health-care workers’ good grief counseling skills. It is likely that a responsible health-care worker will not only devote much attention to a patient’s condition but also show concern for bereaved families. Responsible health-care workers have accumulated abundant experience in the process of receiving bereaved families, which has continuously improved their skills. Next, we found that health-care workers with more experience receiving bereaved families in general are better equipped to offer grief counseling. This result was generally consistent with that of previous research.^19,24^ It is likely that, during frequent contacts with bereaved families, health-care workers not only realized the need for grief counseling but also grasped stronger communication skills. If grief counseling training is carried out in the future, it is recommended that trainers should specifically focus on health-care workers with less experience of receiving bereaved families. Last, health-care workers who have received relevant training have significantly better grief counseling skills. Relevant training included grief counseling, grief therapy, death education, and hospice care.^23^ Luo and Fu’s research also proved that relevant training can help Chinese health-care workers quickly master the theory and practical skills of grief counseling.^17^

### Factors Affecting Attitudes of Grief Counseling Among Health-Care Workers

Our research demonstrated that the potential influencing factors of attitudes of grief counseling are other grief counseling skills, communication skills, education background, and relevant training. First, we found that the stronger their skills of grief counseling health-care workers were, the more positive their attitudes would be. Other grief counseling skills included health education skills, grief risk assessment, psychological interventions, and self-emotional regulation.^23^ This result was aligned with a previous research.^19^ It is likely due to the fact that health-care workers with excellent abilities in grief counseling often have rich practical experience in grief counseling emphasizing strong humanistic care. Therefore, they are more enthusiastic about getting involved in efforts related to bereaved families. Second, we found that health-care workers’ communication skills were closely related to their attitudes of grief counseling. Communication skills included counseling skills, empathy ability, listening ability, and expressive ability.^23^ This is probably because health-care workers with good communication skills can often accurately and rapidly grasp the personality characteristics and psychological condition of bereaved families, and choose the corresponding communication strategy. Third, the higher the education level of health-care workers, the more positive their attitude toward the bereaved. In addition to receiving abundant training as part an advanced medical curriculum, highly educated health-care workers have a better comprehensive quality, which enables them to implement the concept of humanistic medicine in clinical work. Finally, this research showed that after receiving relevant training, health-care workers can clearly improve their attitude toward grief counseling. The relevant training included death education, life education, hospice care, grief counseling, and grief treatment. It is strongly recommended that relevant training or education should be offered to health-care workers as soon as possible.

To this end, the following suggestions are put forth. First, grief counseling is a complex systematic task involving multiple professionals, including health care workers, grief therapists, psychological counselors, psychiatrists, social workers, and family therapists. A hospital-community information system should be established so that health care workers can share the therapeutic process of the deceased with family therapists and social workers. In this way, social workers and family therapists can provide more targeted grief counseling to bereaved families. Second, a standardized training course should be established immediately in China. Because the threat posed by the pandemic remains, online public classes, webcast, and short videos are recommended for training. Health care workers with lower education levels and health care workers with less experience of receiving bereaved families should be paid more attention to in the training. Third, it is necessary to strengthen the promulgation of grief counseling in China. Workers in communities, schools, and hospitals can all be mobilized to publicize and popularize the concept of grief counseling to the public.

### Limitations

Several limitations of this research should be considered. First, as a cross-sectional survey, this research could only assess the status of grief counseling at a specified time without follow-up observations of the health care workers. Second, due the limited number of hospitals that participated in this survey, our sample size might be too small to generalize the results. Thus, it is necessary to carry out a larger scale research in the future. Finally, as AGCS and SGCS are relatively new scales, so they may not have a history of much use. Considering the urgency of the COVID-19, time stresses did not allow us to test these scales fully ahead of use.

## Conclusions

During the COVID-19 pandemic, over 2 million people have lost their lives worldwide.^6^ While the primary focus of the public has been on COVID-19 patients, less attention has been paid to struggles of bereaved families. This study is the first research to focus on Chinese health care workers’ grief counseling for bereaved families of COVID-19 victims. The skills and attitudes toward grief counseling among health care workers combating COVID-19 were at a lower level in Wuhan, indicating the need to urgently improve grief counseling. The main influence factors of grief counseling skills consisted of sense of responsibility, frequency of contact with bereaved families, and relevant training, while the main influence factors of attitudes toward grief counseling consisted of other grief counseling skills, communication skills, education background, and relevant training. China should build a comprehensive grief counseling system, establish a standardized training course, and strengthen the popularization of grief counseling to the public.
